# Reported Acquisition Practices of Australian Dog Owners

**DOI:** 10.3390/ani9121157

**Published:** 2019-12-17

**Authors:** Simone A. Blackman, Bethany J. Wilson, Alistair R. Reed, Paul D. McGreevy

**Affiliations:** 1 Tasmanian College of Business and Economics, University of Tasmania, Hobart, TAS 7005, Australia; 2 Sydney School of Veterinary Science, Faculty of Science, The University of Sydney, Sydney, NSW 2006, Australia; 3 Independent Researcher, Hobart, TAS 7170, Australia

**Keywords:** canine, dog, acquisition, purchase, priorities

## Abstract

**Simple Summary:**

There are more than 205 pure-breeds of dogs and many mixed or cross-breeds available to those seeking to acquire a dog in Australia. It is estimated that 400,000 dogs are acquired annually but there is little information about how prospective owners make their choices and what canine attributes they prioritise when acquiring dogs. Attributes influencing owner’s choice include: physical appearance, behavioural characteristics, breed reputation, how a dog has been bred, perceived genetic soundness, and a dog’s need for rehoming or rescuing. This study considers the choices owners make in selecting their most recently acquired dogs and explores how owners prioritise various attributes. Analysis of survey results suggests that Australian dog owners fall into two distinct groups: one that prioritises the ability to rescue a dog, compatibility on meeting and how compatible they believe the dog will be with their family, and the second that prioritises attributes associated with how the dog was bred including morphotype, how a dog has been raised, genetic testing, and temperament predictability. Each group is making different but not substantially more optimal acquisition choices than one another.

**Abstract:**

In Australia, the UK and the US dog ownership is prevalent with an estimated 40% of Australian households, 25% of UK households, and 50% of US households owning a dog. Once acquired, a dog usually becomes a family companion so, unlike a faulty product, it can rarely be returned or resold without some emotional impact on both the acquirer and the dog. Regarding the reality of dog relinquishment, there is a growing need for cross-disciplinary research that considers how dog owners are making their acquisition choices and, if prioritising different attributes, leads to more optimal acquisition choices. This research collected data from 2840 dog owners via an online survey and examines how owners prioritised various attributes when acquiring their latest dog. The Pearson-Blotchky analysis of survey results show owners are split into two groups, with each group prioritising different attributes or characteristics in their search for a new dog. The first group are those dog owners who prioritised: the ability to rescue a dog, how compatible the dog was on the first meeting, and how compatible they believed the dog would be with their household. The second group are those owners who prioritised: a dog’s morphology, temperament predictability, and breeding practices. While each group prioritised different attributes, neither group made substantially more optimal acquisition choices in terms of overall satisfaction with the dog that they ultimately selected.

## 1. Introduction

It is estimated that around 50% of American households, 40% of Australian households, and 25% of households in the UK currently own a dog [1,2,3]. In these countries, some dogs can change hands for many thousands of dollars (pounds) while others remain in shelters, unable to attract an owner. Irrespective of price paid, most owners view their dog as a companion or family member [4]. Many owners want to acquire a dog that is able to complement their lifestyle, so they seek out dogs that are temperamentally, physically, and genetically sound [5]. Others prioritise different attributes so may prioritise: a dog’s need to find a new home, its perceived fashionableness, owner resemblance, or even associations with celebrities [6].

There is a substantial variation in the median lifespan across various breeds with an average span of approximately 10.5 years [7]. If continuity of dog ownership is assumed, then an owner may acquire more than six different dogs over their own lifetime. In Australia, this equates to an estimated market demand of approximately 400,000 puppies per year. In Australia, 69,336 puppies were recorded as being produced by registered dog breeders in 2018 [8], which is less than 18% of the estimated potential demand. The term ’registered breeder’ in this case means a breeder who is a financial member of the national kennel club in Australia and the UK. In the UK, it is estimated that more than 30% of demand is met by registered breeders [9]. The remainder of puppies required to meet demand in both of these markets must originate as a result of either unintentional breeding (accidents) or by intentional breeding by unregistered breeders.

Importantly, being a registered breeder does not necessarily guarantee good breeding practices or welfare standards despite the aims of breeding codes to which registered breeders might be required to comply. Similarly, while unregistered breeders may not be held accountable against codes of conduct or best practice, this alone does not imply poor breeding practices.

There are several options available to dog seekers. Prospective owners may approach a registered hobby or a commercial pure-breed or cross-breed breeder. In addition, they may acquire a dog from a shelter, rescue organisation, or from an occasional or backyard breeder. The acquisition price varies but almost all dogs come at some financial cost to a prospective owner. Similarly, the amount and quality of information available to prospective owners, which might inform their decision to acquire, may also vary. There is no standard for how a breed or type of dog might be presented to a prospective owner. Breeders and dog sellers, like owners, are individually motivated. Some dog sellers may be interested in a particular breed, emphasising the benefits of that breed without necessarily informing the prospective owner of potential problems (for example, potential health issues in a breed or incompatibility of a type of dog with an owner’s lifestyle). Other breeders and third-party sellers might be driven more by profit and be keen to sell, irrespective of the potential buyer’s suitability as a dog owner [10,11]. Not all dogs being acquired are sold by their breeders. The reality is that many dogs are either sold by third party sellers or by shelters and rescue organisations. These shelters and rescue organisations are tasked with the role of finding suitable homes for dogs that have been either abandoned or relinquished. Many of these shelters and rescue organisations may hold little information about the dog’s history. Potential purchasers may or may not be aware that they are making informed acquisition choices.

There is an increasing awareness of the roles that dogs play in the lives of their owners. Dog owners may list many reasons for acquiring a dog and there is increasing research that explores why owners acquire dogs and the associated benefits and challenges of dog ownership [12]. Benefits that dogs may provide include providing support, companionship, social engagement [13], and increased physical activity [14]. Challenges faced by dog owners include, for example, meeting the financial costs of owning a dog. While some owners see their dog as an object, others may view their dog as a companion or even as a respected significant other [15]. Beverland et al. showed that some owners are intrinsically motivated while others are extrinsically motivated. Those who are intrinsically motivated may value their dog for its own sake. Those who are more extrinsically motivated seek external validation and so might prefer a dog with a specific appearance to gain social acknowledgement [16]. This framework is supported by Bettany et al., which confirms that some owners value their dogs as a mechanism for improving their social status [17].

There is little published research that considers how people prioritise attributes and characteristics when acquiring a dog. One recent Danish study examined puppy buyers’ motivations when purchasing a puppy with known inherited disorders [6]. Participants for this study were selected at random from a list of owners within the Danish Dog Registry, which is a body established in 1993 when the Danish Government made it compulsory for all dogs in the country to have an ID number and to be recorded on this register [18]. The study randomly generated a list of 750 owners of each breed and the researchers sent a letter and questionnaire to these 3000 owners. Just more than 35% (*n* = 911) of owners returned the completed questionnaire. This study considered the motivations that drove the selection by owners of four different pure-breeds. Importantly, it did not consider the acquisition choices that apply to dog owners in general, irrespective of breed. The study found that, when considering the purchase of a Cavalier King Charles Spaniel or Cairn Terrier, respondents were concerned mainly about breed attributes, such as health. By contrast, those seeking to acquire a French Bulldog prioritised appearance and personality, while those seeking to acquire a Chihuahua prioritised ease of procurement [6]. This study also revealed that caregiving behaviour increase the likelihood of the purchase of dogs with infantile facial features such as large foreheads, large and low-lying eyes, and bulging cheeks [5].

Weiss and others (2012) [19] asked recent animal adopters (*n* = 1491) to rank the importance of a number of characteristics that they could observe about an animal prior to selection and information that they received prior to selection. Participants ranked the importance of observing the animal’s behaviour along with the information that they received about the animal’s behaviour, health, and history. The most important reason a dog was chosen was its appearance (27.3% of survey participants indicated that this was the single most important reason), followed by its personality/temperament (15.8%), and, thirdly, how it behaved with people (11.4%) [19]. This study focused solely on dogs acquired from shelters.

A 2009 Australian study surveyed 877 potential or actual dog owners, asking them to rank statements about their ’ideal’ dog [5]. The study concluded that certain physical and behavioural characteristics were prioritised and that the ideal Australian dog is:

*De-sexed, has short/straight hair, is of medium size (10–20 kg), is acquired as a puppy, and requires between 16-min and 30-min exercise per day and between 1-min and 15-min grooming per week. The “ideal dog” is also safe with children, housetrained, healthy, comes when called, does not escape the property, is not destructive when left alone, lives until at least 10 years old, and is obedient, friendly, and affectionate*.

[5]

A 2016 US study considered the online responses provided by 669 dog-owning households around spending behaviours and dog acquisition practices [20]. This confirmed that 47% of owners acquired the dog that they did because it was the ’right thing to do.’ Furthermore, 33% of participants confirmed they wanted a specific breed of dog and 22% relied chiefly on previous experience to guide their selection. The influential characteristics were a dog’s compatibility with owner lifestyle (60%), the dog’s behaviour (58%), and physical health (50%).

A comprehensive review [11] summarised owners’ decisions to acquire a dog, what type of dog, which individual dog, and the source of the dog. It confirmed the need for more cross-disciplinary research into the factors and attributes that motivate and influence prospective owners when making their acquisition choices [11]. Furthermore, it noted that much of the research previously undertaken considers the intentions of would-be dog owners instead of the behaviour of actual dog owners as they make their selection decisions and largely focuses on anticipated acquisition from shelters [11]. More research is needed to reveal the actual acquisition practices and behaviours undertaken by dog owners, irrespective of the anticipated source.

The current study was designed to explore the attributes that Australian dog owners prioritised when acquiring a dog and to consider which acquisition attributes, if any, can be considered ’best practice’ acquisition choices in terms of eventual owner satisfaction. Understanding the attributes dog owners prioritise when acquiring dogs and which of these lead to maximal post-acquisition satisfaction will facilitate policy decisions around dog regulation and assist those who sell and rehome dogs.

This research study considered the following questions. First, what do dog owners prioritise when making acquisition choices? Second, what is the distribution of best practice (i.e., the prioritisation of dog attributes at acquisition that provided an owner with the most satisfaction when owning a dog) and, third, are those that acquire pure-breed dogs more or less satisfied with their acquisition choices than those acquiring cross-breed dogs?

## 2. Methods

### 2.1. Survey Design and Materials

The survey consisted of 30 questions grouped into three sections. Participants were not required to answer all questions. They were asked to supply details of their most recently acquired dog. Questions investigated included the amount of research undertaken prior to acquisition, place of acquisition, price paid, breed type or breed, type of information supplied by the seller, level of ongoing contact with the breeder/seller, role that the dog plays in their life, and overall satisfaction level with the dog. Participants were also asked to provide data on their gender, age, residency, and the highest level of education obtained.

Survey participants were asked to rank the importance of attributes of their most recently acquired dog. Specifically, owners were asked to score the following 11 options as: not important (1), somewhat important (2), important (3), very important (4), or essential (5).

- That the dog was of a specific breed;
- That the dog had a good temperament when the respondent first met him or her (temperament on meeting);
- That the respondent could meet the breeder and/or view the parents of the dog (view parents);
- How the puppy/dog had been raised (husbandry);
- Predictability about size at maturity (size predictability);
- Predictability about temperament and future needs (temperament predictability);
- That the respondent was able to rescue/house a dog that needed a good home (rescue/homing);
- That the dog was bred by a registered member of a canine association (registered breeder);
- That the dog would get on with other animals and/or children (compatibility with household);
- That the dog had good conformation/structure (conformation);
- That the dog had been genetically tested by its breeder for potential genetic issues (genetic testing);

Survey participants were questioned about their past dog seeking behaviour and are referred to as dog seekers. However, at the time of the survey, participants were current dog owners. This is recognised and discussed below as a possible limitation of this work.

The questionnaire concluded with items to elicit general demographic information.

### 2.2. Procedure

The Human Research Ethics Committee (Tasmania) Network (Approval No. H0013192) approved this research. The survey was administered online and was anonymous, unless participants elected to provide contact details. Positive consent was obtained from all respondents.

### 2.3. Survey Distribution

The survey was open online for eight weeks (from 11 May 2015 to 6 July 2015). It was conducted using commercial survey software (Survey Monkey, San Mateo, CA, USA) and was promoted in several ways. Web-links were created for distribution on social media and Facebook groups. Postcards setting out the aim of the study and listing a web-link were distributed at a number of dog events, such as at dog-walking meetings and the *Million Paws Walk* in Tasmania, which is an annual fundraising event undertaken by the Royal Society for the Prevention of Cruelty to Animals (RSPCA) across Australia [21]. Cards were also posted to 40 veterinary practices and dog-grooming businesses randomly chosen across Australia.

### 2.4. Statistical Analysis

The data that were obtained from the questionnaire were downloaded into Excel spreadsheets and into R statistical and computing software for analysis [22]. Patterns in the priorities of questionnaire respondents were initially assessed using Kendall’s Tau metric of association. Following this, hierarchical cluster analysis was performed using the hclust () function on a Minkowski distance matrix [22]. The identified clusters of respondents were assessed for demographic differences in gender and age using χ^2^ tests. Differences in the likelihood of purchasing a pure-breed dog among identified clusters were also assessed using χ^2^ tests. Differences in residency and education using the non-parametric Wilcoxon Rank Sum Test were assessed. Satisfaction in purchasing decisions between the identified clusters were also assessed by the Wilcoxon Rank Sum Test.

## 3. Results

### 3.1. Response Rates

A total of 2840 Australian dog owners completed the survey. The relative response rate could not be calculated because the survey was distributed using postcards and social networks to sample a population of an unknown number. Since this research sought to consider acquisition practices, a decision was made to exclude data from those who bred their own dogs. This was done by excluding 682 participants who answered ’Yes’ to a question in the survey that asked them to indicate if they were a member of a State or Territory Canine Association. This recognised that a large proportion of this group of participants likely bred rather than acquired their dog. This left a total of 2158 participants.

### 3.2. Acquisition Choices

Participants were asked to recall the last dog that they had acquired and report on the importance that they had placed on the 11 attributes when they made their acquisition decision. Participants did this by deciding if each of the listed options was considered by them to be essential, very important, important, somewhat important, or not important. 

**Figure 1:**
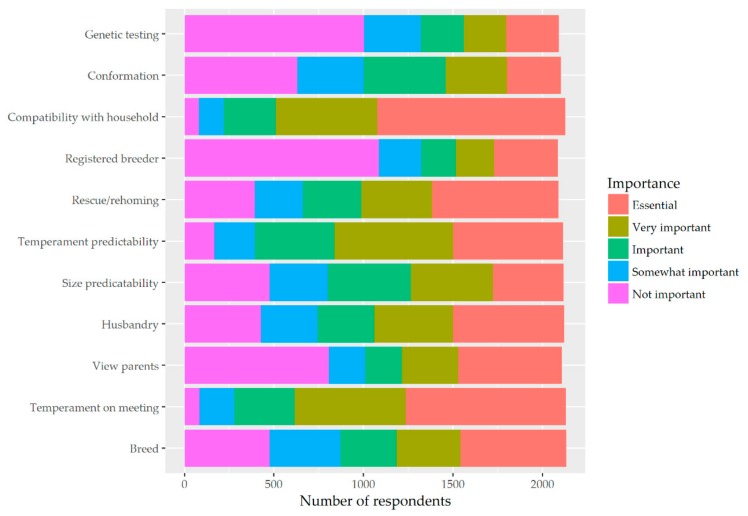
Attributes of dogs, their parents, and their carers/breeders that are prioritised by owners.

Figure 1 provides a colour-coded representation of the responses to each of these attributes. The least important considerations were, firstly, whether the dog had been bred by a registered breeder (52.1% indicating that this was not important) and, secondly, whether the parents of the dog had undergone genetic testing (48.0%). The most important considerations were that the dog being considered had a good temperament on meeting and that it would have compatibility with the household. Additionally, 76% of participants indicated that compatibility with the household was either essential or very important and only 3.8% indicated that this was not important. Similarly, 70% of participants indicated that temperament during the first meeting was either an essential or very important attribute and only 4.0% indicated that it was not at all important. When asked to consider the importance of the breed, more than a quarter of all participants who answered this question indicated that breed was an essential characteristic (27.6%), but 22.3% indicated that it was not important.

### 3.3. What Do Dog Owners Prioritise When Making Acquisition Choices?

**Figure 2:**
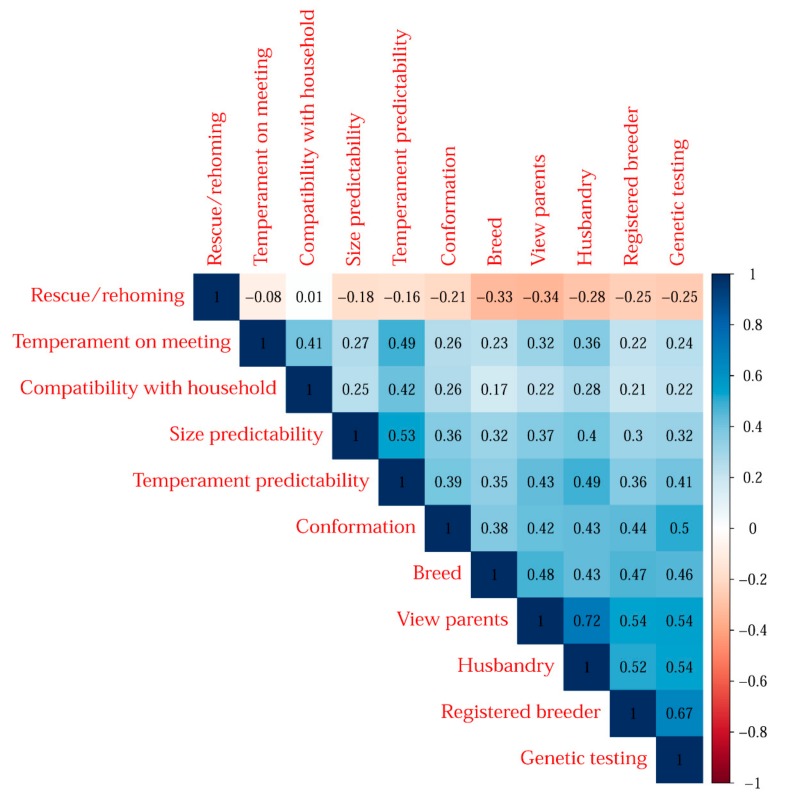
Kendall’s Tau associations between the priorities that respondents reported when acquiring a dog. The blue squares show positive associations between attributes and the red squares show negative associations. The darker the colour is, the stronger the association is. This does not mean that participants have responded positively or negatively to any one attribute but rather that survey participants have responded with similar levels of importance between attributes. The ’tipping point’ is the point at which no correlation can be shown in the data (no colour) between attributes, which is most closely represented between the importance attributed by survey respondents to a dog’s compatibility with the household (Attribute 1), and the need to rescue/home a dog (Attribute 2).

Figure 2 shows these Kendall’s Tau associations between priorities when making acquisition choices, which shows the patterns in the priorities.

There were strong correlations between many of the attributes that a dog seeker might encounter when considering an acquisition from a registered breeder. For example, genetic testing is likely to be important to a buyer wanting to acquire a pure-breed dog. Conversely, those who are more interested in rescue/rehoming a dog are less likely to be interested in the genetic testing of that dog or the ability to view parents. In general, dog owners who prioritise rescue/rehoming a dog tend to place less emphasis on attributes most commonly associated with dogs acquired from registered breeders. Conversely, the data show that those interested in acquiring a particular breed of dog will prioritise genetic testing, a registered breeder, husbandry, and the view of parents.

**Figure 3:**
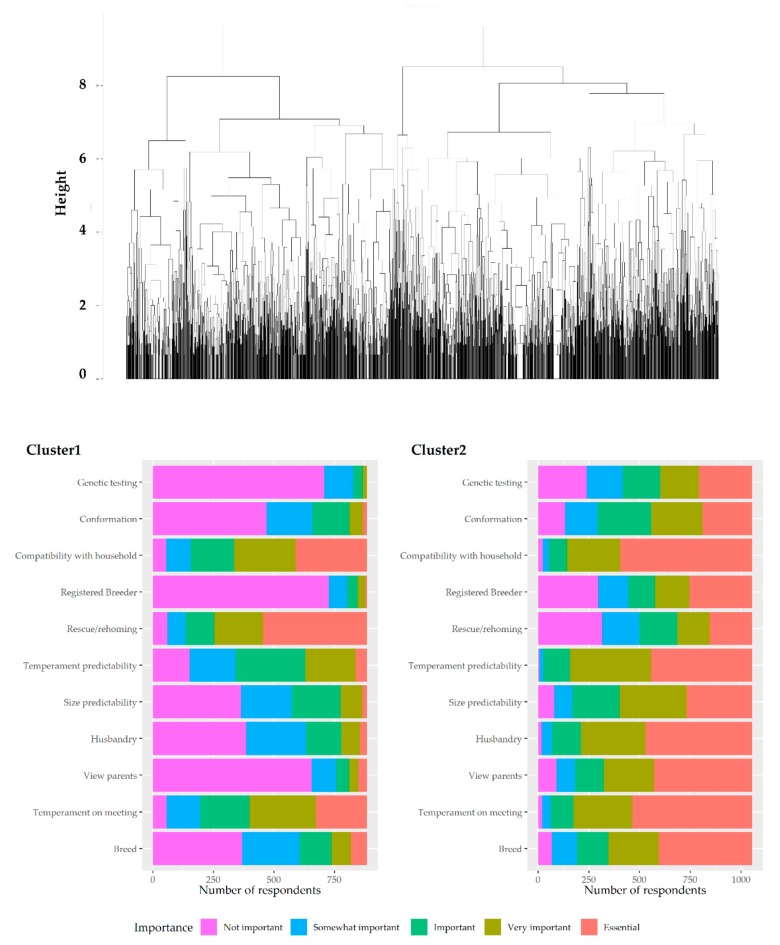
Cluster dendrogram and cluster analysis of attributes as prioritised by dog owners.

Figure 3 shows a cluster dendrogram and cluster analysis of attributes as prioritised by survey participants to show the relationships among the scores provided by participants on the importance of the 11 attributes.

Having considered the data set out in Figure 1, Figure 2 and Figure 3, clear patterns and associations between attributes emerge and two groups of dog seekers become apparent. The more dog seekers prioritise rescue/homing, the less they prioritise other aspects (but especially breed, view of parents, and husbandry). Conversely, another cluster emerges. These are dog seekers who prioritise genetic testing and who also tend to prioritise the importance of acquiring a dog from a registered breeder. Having seen these clusters emerge, a decision was made to divide responses into these two clusters and to examine the different priority patterns of these two distinct groups of dog seekers. 

The first group of dog seekers (Cluster 1, Figure 3) are those who prioritise a rescue (R) dog and who are mainly looking at their temperament on the first meeting (T) and their perceived compatibility with their household (C). This paper calls this group *RTC seekers*. The second group or cluster, as shown in Figure 3 as Cluster 2, are those who are looking for a particular morphotype (M) of dog, and, in addition to compatibility with the household and temperament on the first meeting, they appear to be looking to support those that they believe to be good breeders and characteristics associated with them (optimal breeding practice (OBP): registration, good puppy husbandry, ability to view parents, genetic testing, and temperament predictability). This paper calls this group *MOBP seekers*.

### 3.4. The Distribution of Best Practice in Dog Acquisition in Terms of Owner Satisfaction

Dog owners were asked how satisfied they were with their purchasing/acquisition choices in relation to their most recently acquired dog. Owners were asked to rank their satisfaction from being *very unsatisfied, not satisfied, somewhat satisfied, and very satisfied* to *completely satisfied*. Table 1 sets out the results of dog seeker satisfaction of all participants and of the RTC and MOBP seekers.

Table 1 reveals that fewer than 90% of participants were either very satisfied or completely satisfied with their acquisition choice. This table also reveals that MOPB seekers are significantly more satisfied with their acquisitions than RTC seekers. Furthermore, 90.9% of MOBP seekers were either very satisfied or completely satisfied with their acquisition choice while 87.3% of RTC seekers were either very satisfied or completely satisfied with their choice.

### 3.5. Are Those Who Acquire Pure-Breed Dogs More Satisfied with Their Acquisition Choices than Those Who Acquire Cross-Breed Dogs?

Respondents were asked if they had acquired a pure-breed dog. In addition, 900 participants stated ’No’ (42.2%) and 1233 answered ’Yes’ (57.8%). Table 2 shows the type of dog acquired by the two clusters of dog seekers.

Table 2 shows that MOBP seekers were significantly more likely to purchase a pure-breed dog than RTC seekers. Approximately three-quarters of all MOBP seekers had acquired what they believed to be a pure-bred dog whereas only 37.8% of RTC believed that the dog that they had acquired was a pure-breed.

Next, the satisfaction of those who purchased/acquired a pure-bred dog was compared to the satisfaction of those who acquired a cross-bred dog.

Table 3 shows little difference in the aggregate percentages for those expressing positive satisfaction in purchasing either cross-breed or pure-breed dogs. However, a Wilcoxon Rank Sum test shows that those who purchased pure-breed dogs were significantly more satisfied than those who purchased cross-breeds (*p* = 0.02).

### 3.6. Demographics of Dog Seekers

Responses to the demographic survey questions permitted an assessment of any possible differences between the MOBP seekers and RTC seekers based on gender, age, residency, or education. The results appear in Table 4, Table 5, Table 6 and Table 7.

Table 4 shows that of the MOBP seekers 89.9% (868) were female and 10.1% (98) were male. Of RTC seekers, 88.2% (721) were female and 11.8% (96) were male. There is little difference in the ratio of women in the two clusters of dog seekers, other than there being a slightly higher proportion of men in the RTC seekers cluster than in the MOBP seekers cluster. 

Table 5 reveals little difference in age between RTC seekers and MOBP seekers.

Table 6 shows slightly more MOBP seekers were born in Australia than RTC seekers, with just more than 20% of RTC seekers having been born overseas and only 16.2% of MOBP seekers having been born overseas.

Table 7 indicates little difference in the levels of education by RTC and MOBP seekers. Based on these results, levels of education do not appear to be a deciding factor in owner satisfaction.

## 4. Discussion

The primary research question focused on what dog owners prioritise when making acquisition choices. In alignment with previous reports [20], the current findings indicate that owners place varying importance on attributes that dogs have when they acquired their most recent dog. Unlike previous research that reported on potential choices of dog owners by asking them to indicate the most and least ethical sources from which to acquire a dog, the current study asked participants to indicate where they had obtained their most recently acquired dog and to rank various attributes, according to their importance during the acquisition decision [23].

The most important attribute to survey participants, as set out in Figure 1, was compatibility with their household, which is closely followed by temperament during the first meeting. Interestingly, these two attributes show a correlation of 0.41 suggesting survey participants are less likely to rank these attributes equally than, for example, genetic testing and the dog being acquired from a registered breeder, which had one of the highest correlations of 0.67. This suggests that those seeking to purchase a pure-bred dog expect that its registered breeder will have undertaken the necessary genetic testing. There was a positive but weak correlation between those ranking compatibility with household and its temperament on the first meeting. Owners regarded temperament predictability and size predictability as equally important, with these two attributes having a correlation of 0.53. 

The highest correlation was between the attributes of viewing the parents and the importance of husbandry (0.72). This suggests that dog seekers who are interested in how a dog has been reared are almost certainly going to be as interested in seeing its parents. Conversely, those who are not interested in its husbandry will not deem it important to view its parents.

There is a strong positive correlation (0.72) between those who wanted to be informed about a dog’s husbandry (how it was raised) and a need to see its parents. This is in contrast to those who prioritised rescuing a dog over viewing its parents (−0.034). This suggests that people interested in meeting the breeder and/or viewing the dog’s parents feel more strongly about their need to know and see how a dog is raised than those who want to rescue a dog. What has emerged from the current study is the existence of two distinct clusters of dog seekers. First, there is the group that this report calls the RTC seekers. These are owners who prioritised the importance of rescuing or rehoming a dog above any other single attribute. This research confirmed that it was either essential or very important to them when considering the acquisition that they were able to rescue/rehome a dog that needed a good home. This finding is consistent with research by Sandoe et al., which found that some owners actively seek out dogs with attributes that suggest that they need care, such as Chihuahuas that are small and, therefore, in need of ’help’, over and above any other attribute of the dog [6]. This research also found that these respondents also sought out a dog that they believed would be compatible with their household and one that had a suitable temperament on the first meeting. This group were less concerned about meeting the dog’s breeder or parents and about genetic testing.

The second group that emerged are those seekers who place high importance on breed type, predictability of temperament, and on how the dog had been raised. This group of seekers, whom this research calls MOBP seekers, look for a particular morphotype of dog and place great importance on compatibility with the household and temperament on the day of the first meeting. They appear to support good breeders and characteristics associated with them, notably an optimal breeding practice. These characteristics include breeder registration, good puppy husbandry, ability to view parents, genetic testing, and predictability of temperament.

It is not possible to say which cluster of dog seekers exhibit better acquisition practices. In common, both groups prioritise compatibility with their household. The RTC seekers also prioritise rescuing and rehoming, and, thus, may exhibit a clear desire to do what they believe is the moral or right thing. This aligns with Bir et al. who reported that many seekers prioritise rescuing or rehoming and ’doing the right thing’ [20]. The ’rightness’ of this is not absolute as a counter-view may be that, while admirable, it is a practice that might lead to disappointment should the dog not live up to expectations. This study reveals RTC seekers are slightly less satisfied with their actual acquisition than MOBP seekers.

The question about trading off ’doing the right thing’ with a given level of satisfaction is a decision that is ultimately for each individual owner to make. Where the trade-off becomes problematic is if an owner acquires a dog that is not a good fit. This may lead disappointed RTC seekers to return the dog to its source or into a rescue or shelter organisation. Thus, there is a trade-off between more future-focused attributes, such as husbandry, against the ability to take on a dog that meets a dog owner’s need to give care [6]. MOBP seekers prioritise more future-focused attributes, which are ones more geared to ensuring that the dog is temperamentally, physically, and genetically sound and, therefore, perhaps more likely to become a better fit with their lifestyle and more able to provide them with other benefits, such as social acknowledgement and status [16,17]. The MOBP seekers will believe that the way that they prioritise attributes during acquisition is both optimal and the right way to go about a search for their next dog. Their choices support registered breeders who ideally embrace optimal breeding practices such as good husbandry and genetic testing that ensure their puppies are physically sound and behaviourally predictable. These buyers may believe that, by acquiring a pure-breed dog, that they support ongoing stewardship of existing breeds. However, by seeking to purchase a specific breed, they may also sustain the breeding of dogs with inherited disorders.

Despite the differences that emerge between the two clusters of dog seekers, it is clear from the current study that both groups place considerable importance on temperament during the first meeting and compatibility with the household. This is consistent with the report by Weiss et al. [19] that found that, second only to the dog’s appearance, its personality and temperament were the most important reasons people chose the pet they did. It is consistent with the findings of King et al. who revealed that Australians favour dogs that are safe with children, friendly, and affectionate [5]. This research confirmed that, regardless of its source of acquisition, it is important for a dog to have a good temperament and be compatible with the household it will be joining. The wider implication of this in terms of re-homing rates is that, irrespective of where dogs are being bred and how they are being acquired, owners will be more satisfied with dogs, and, therefore, more likely to retain a dog that has a good temperament and is compatible with their household. Those who home dogs and those who promote good breeding practices should be mindful of this if they want to improve retention and re-homing rates.

This study revealed little difference in the gender, age, residency, or education levels of the two clusters. The reality of dog acquisition practices may be that a dog is being acquired by a household, not just by one member of the household.

A number of limitations of this study are recognised. The primary limitation is that participants were current dog owners at the time that they completed the survey and they were asked to report on their recollections of their dog seeking processes. It is recognised that their motives for seeking a dog may have changed through the buying process and their recollections may not be accurate. Another limitation is that some dog owners did not actively acquire a dog. They may have gained a dog through inheritance or in some other way, so that they did not deliberate over the dog’s attributes at the time of acquisition. Furthermore, data from survey participants who answered ’Yes’ to being a member of a canine association were excluded. This was done to exclude breeders, recognising that dog enthusiasts may not describe themselves as breeders. On this basis, a decision was made to use membership of canine associations as the best proxy for recognising participants as breeders. A future study could be undertaken to interrogate the data from the 682 participants who were excluded from the current analysis. The final limitation is that dog owners were asked to report the age of the dog at the time of the survey and not at the time of acquisition. Therefore, there was no way to test whether age of dog at acquisition had a bearing on the importance of attributes such as the need for genetic testing and viewing the parents. Research that explores whether age of a dog at acquisition correlates with other acquisition attributes would be useful.

Along with the future study identified above, future research is needed to determine if the results of the current cross-sectional study hold true over time. A longitudinal study assessing dog owners’ satisfaction over time is needed to obtain a deeper understanding of the links between acquisition practices and owner satisfaction.

## 5. Conclusions

The current study examined the acquisition choices dog owners make in Australia and owners’ satisfaction with the dogs that result from those choices. Through the examination of acquisition choices, two groups of dog owners emerged with different perspectives on the importance of various attributes when acquiring their dog. These groups were RTC seekers, who prioritised the ability to rescue a dog, temperament during the first meeting, and perceived compatibility with their household while MOBP seekers prioritised morphology and what they perceived to be optimal breeding practices are satisfied. The current study found that more than 96% of each group are either somewhat satisfied, very satisfied, or completely satisfied with their choice of dog. What difference emerged is that the MOBP seekers are more satisfied overall with their purchasing decision than RTC seekers (*p* = 0.02).

## Figures and Tables

**Table 1:** Satisfaction with acquisition across all participants.

Level of satisfaction	No Answer Provided	Very Unsatisfied	Not Satisfied	Somewhat Satisfied	Very Satisfied	Completely Satisfied	Mean
	*n* (%)	*n* (%)	*n* (%)	*n* (%)	*n* (%)	*n* (%)	
**All participants**	17 (0.8)	26 (1.2)	30 (1.4)	156 (7.2)	359 (16.6)	1570 (72.7)	
**RTC seekers**	8 (0.9)	10 (1.1)	12 (1.4)	82 (9.3)	156 (17.6)	617 (69.7)	4.548461
**MOBP seekers**	6 (0.6)	14 (1.3)	14 (1.3)	62 (5.9)	172 (16.3)	787 (74.6)	4.624404

RTC seekers: dog seekers who prioritise a rescue (R) dog and who are mainly looking at their temperament on the first meeting (T) and their perceived compatibility with their household (C). MOBP seekers: dog seekers who are looking for a particular morphotype (M) of dog, and, in addition to compatibility with the household and temperament on the first meeting, they appear to be looking to support those that they believe to be good breeders and characteristics associated with them (optimal breeding practice (OBP): registration, good puppy husbandry, ability to view parents, genetic testing, and temperament predictability).

**Table 2:** Types of dog acquired by the two clusters of dog seekers.

Type of Dog Acquired	RTC Seekers	MOBP Seekers
	*n* (%)	*n* (%)
Cross-breed	544 (62.2)	269 (25.8)
Pure-breed	331 (37.8)	774 (74.2)
**Total**	**875**	**1043**

**Table 3:** Levels of satisfaction reported by pure-breed acquirers versus cross-breed acquirers.

Type of Dog	Unsatisfied		Satisfied			
	Very Unsatisfied	Not Satisfied	Somewhat Satisfied	Very Satisfied	Completely Satisfied	Total Satisfied
	*n* (%)	*n* (%)	*n* (%)	*n* (%)	*n* (%)	%
**Cross-breed**	9 (1.1)	12 (1.4)	79 (9.3)	133 (17.6)	573 (69.7)	96.6
**Pure-breed**	14 (1.3)	14 (1.3)	65 (5.9)	190 (16.3)	816 (74.6)	96.8

**Table 4:** Gender distribution between the clusters of dog seekers.

Cluster of Seeker	Female	Male	Total
	*n* (%)	*n* (%)	*n* (%)
**MOBP seekers**	868 (89.9)	98 (10.1)	966
**RTC seekers**	721 (88.2)	96 (11.8)	817

**Table 5:** Age of the clusters of dog seekers.

Cluster of Seeker (Ages)	Age of Respondents								
	Under 18 Years	18 to 24 Years	25 to 34 Years	35 to 44 Years	45 to 54 Years	55 to 64 Years	65 to 74 Years	75 Years or Older	Total
	*n* (%)	*n* (%)	*n* ($%)	*n* (%)	*n* (%)	*n* (%)	*n* (%)	*n* (%)	
**RTC seekers**	6 (0.7)	54 (6.6)	180 (22)	199 (24.4)	234 (28.6)	112 (13.7)	25 (3.1)	7 (0.9)	817
**MOBP seekers**	7 (0.7)	66 (6.8)	223 (23.1)	236 (24.4)	254 (26.3)	144 (14.9)	35 (3.6)	2 (0.2)	967

**Table 6:** Residency of the clusters of dog seekers.

Cluster of Seeker (Residency)	Born in Australia	Been Here Over 10 Years	Been in Australia Less Than 10 Years	Total
	*n* (%)	*n* (%)	*n* (%)	
**RTC seekers**	639 (79.9)	130 (16.3)	31 (3.9)	800
**MOBP seekers**	798 (83.8)	124 (13.0)	30 (3.2)	952

**Table 7:** Respondents’ education level across the two clusters of dog seekers.

Cluster of Seeker (Education)	Grade 10	College/Senior Secondary School	Diploma	Associate Degree	Bachelor Degree	Graduate Degree	Post Grad Degree	Total
	*n* (%)	*n* (%)	*n* (%)	*n* (%)	*n* (%)	*n* (%)	*n* (%)	
**RTC seekers**	57 (7.0)	160 (19.6)	134 (16.4)	20 (2.5)	206 (25.3)	54 (6.6)	184 (22.6)	815
**MOBP seekers**	47 (4.9)	187 (19.4)	191 (19.8)	23 (2.4)	221 (22.9)	54 (5.6)	240 (24.9)	963
